# Clinical evaluation of cetuximab combined with an S-1 and oxaliplatin regimen for Chinese patients with advanced gastric cancer

**DOI:** 10.1186/1477-7819-12-115

**Published:** 2014-04-23

**Authors:** Zhan-Dong Zhang, Ye Kong, Wei Yang, Bin Zhang, Yong-Lei Zhang, Er-Min Ma, Hong-Xing Liu, Xiao-Bin Chen, Ya-Wei Hua

**Affiliations:** 1 Department of General Surgery, Affiliated Tumor Hospital of Zhengzhou University, Henan Cancer Hospital, 127 Dongming Road, Zhengzhou 45008, China; 2 Surgical Oncology, The Fifth People’s Hospital of Zhengzhou, 33 Huanghe Road, Zhengzhou 45000, China; 3 Department of Internal Medicine, Affiliated Tumor Hospital of Zhengzhou University, Henan Cancer Hospital, 127 Dongming Road, Zhengzhou 45008, China

## Abstract

**Background:**

The prognosis of patients with advanced gastric cancer is poor. The goal of this study was to evaluate the efficacy and safety of combination therapy of cetuximab and S-1 combined with oxaliplatin (SOX) in Chinese patients with advanced gastric cancer.

**Methods:**

For patients in the experimental group (cetuximab in combination with SOX (Ce-SOX), 30 patients), once-weekly cetuximab (400 mg/m^2^ at the first infusion then 250 mg/m^2^ every week) was administered. For patients in both the control (SOX alone, 26 patients) and experimental groups, oxaliplatin (100 mg/m^2^) was administered intravenously on day 1, while S-1 (80 mg/m^2^/day) was given orally twice daily for 14 days. The endpoints of this study included progression-free survival, response rate, and disease-control rate.

**Results:**

There was no statistically significant difference in response rate between the Ce-SOX and SOX groups (54.8% versus 44%, *P* = 0.225). The difference in disease-control rate was also statistically insignificant between the two groups (87.1% versus 76%, *P* = 0.162). Median progression-free survival in the Ce-SOX group was significantly higher than that in the SOX group (12.8 versus 10.1 months, *P* = 0.007). The median overall survival of the Ce-SOX group and SOX group was 14.0 and 12.2 months, respectively (*P* = 0.043). The one-year survival rate for the Ce-SOX group was 57% compared to 40% in the SOX group. There was no statistical difference in the grade 3 or 4 adverse effects between the two groups.

**Conclusions:**

These findings suggest that the cetuximab combined with SOX regimen is feasible and shows promising efficacy with tolerable adverse effects in Chinese patients with advanced gastric cancer.

## Background

Gastric cancer (GC) is the second leading cause of death from malignant tumor worldwide [1-3], and more than 40% of the world GC cases occur in China [4]. Takahashi *et al*. [2] reported that there were approximately 989,000 new cases of GC and 738,000 deaths worldwide. Increasing early diagnosis of GC has improved the survival rate. However, the prognosis for recurrent or unresectable GC remains poor [2]. Various chemotherapy regimens have been developed for advanced gastric cancer (AGC) patients based on the understanding that chemotherapy can increase the length and quality of life [5].

S-1 is an orally active derivative of 5-FU, which is a fourth generation oral fluoropyrimidine [3,6], and the drug has been used instead of 5-FU in some clinical trials [3,7,8]. Oxaliplatin is typically administered with fluorouracil and leucovorin in a combination known as FOLFOX for the treatment of GC [5]. Previously, clinical studies have reported that S-1 in combination with oxaliplatin has a high response rate ranging from 53 to 59% and an excellent toxicity profile in the treatment of advanced gastric cancer [9-11]. Recently, two phase II trials have proven the efficacy and safety profile of the SOX regimen as a first-line treatment for advanced gastric cancer in Korean and China patients. Patients enrolled in these studies exhibited favorable efficacy and tolerance [12,13].

Cetuximab is a recombinant, human/chimeric IgG1 monoclonal antibody (mAb) directed against the epidermal growth factor receptor (EGFR) [14]. Cetuximab has been proven to be effective as a single agent, improving overall survival (OS) in metastatic colorectal cancer [15] and providing meaningful benefit in advanced head and neck cancer [16]. Cetuximab binds to the extracellular domain of EGFR, thus leading to inhibition of its downstream signaling. EGFR is a transmembrane tyrosine kinase receptor that, on ligand binding, triggers two main signaling pathways, including the RAS-RAF-MAPK and the PI3K-PTEN-AKT pathway [17].

Cetuximab has been evaluated in many phase II studies in patients with advanced GC either as monotherapy or combined with chemotherapy. In the first-line metastatic setting, several trials have evaluated combinations of cetuximab with different chemotherapy regimens [18-22] and all have reported encouraging objective response rates (ORRs) of 40 to 69% and median overall survival (OS) of 9.5 to 17 months. Toxicities have generally been consistent with the additive toxicities of cetuximab and the respective chemotherapy regimens. Grade 3/4 toxicities included neutropenia in 6 to 46% of patients, diarrhea in 4 to 33% of patients, skin toxicity in 6 to 24% of patients, and anaphylaxis to cetuximab in <5% of patients. Furthermore, the combination of cetuximab and FOLFOX4 is effective and well tolerated for AGC [23].

Epidermal growth factor receptor has been found to be overexpressed in gastric cancer [24,25]. Although colorectal tumors with an activating mutation of the Kirsten(K)-ras gene are not sensitive to EGFR antibodies [26], the incidence of K-ras mutations in gastric cancer appears to be low [27-29]. However, no clinical trial has investigated the effect of cetuximab combined with SOX regimen for Chinese patients with AGC. In our study, we investigated the efficacy and safety of cetuximab plus SOX therapy in Chinese patients with AGC.

## Methods

### Patients

Fifty-six patients with histologically or cytologically proven unresectable gastric cancer or recurrence post-operation were enrolled. All of the patients had at least one measurable lesion by the Response Evaluation Criteria in Solid Tumors (RECIST) criteria, were ECOG performance status (PS) 0 to 2 and had anticipated life expectancies >3 months. Additionally, the patients were required to be chemotherapy naive or more than 6 months past the last adjuvant chemotherapy and to possess favorable bone marrow reservation (hemoglobin ≥80 g/L, platelet count ≥100 × 10^9^, leukocyte count = 3 to 10 × 10^9^, neutrophil count ≥1.5 × 10^9^). Laboratory examination showed adequate liver function (total bilirubin ≤1.5 times the upper limit, ALT/AST ≤2.5 times the upper limit) and renal function (blood creatinine ≤ 1.5 mg/dL, creatinine clearance ≥50 mL/min). All patients provided written informed consent and this study was approved by Institutional Review Board of Henna Cancer hospital. All eligible patients were enrolled in this study between 1 August 2010 and 30 September 2012, and were randomly assigned into either the control group (26 patients) or the experimental group (30 patients).

### Treatments

For patients in the experimental group, once-weekly cetuximab (400 mg/m^2^ at the first infusion then 250 mg/m^2^ every week) was administered. For patients in both the control and experimental groups, oxaliplatin (100 mg/m^2^) was administered intravenously on day 1. S-1 (80 mg/m^2^/day) was given orally twice daily for 14 days. All patients then took one week’s rest before the next cycle. Physical examination and blood analysis were performed at each cycle during chemotherapy. Responses of chemotherapy were assessed according to the RECIST criteria. Responses were classified as complete response (CR), partial response (PR), stable disease (SD), and progressive disease (PD). Response rate was calculated as the sum of CR and PR. The disease-control rate was the sum of CR, PR, and SD. Six cycles of chemotherapy were given to all patients who showed response. Computed tomography and magnetic resonance imaging were used to assess tumor size. Second-line chemotherapy was given to patients who did not show response to the SOX or SOX + cetuximab (Ce-SOX) treatment. For grade 3/4 adverse events, the chemotherapy dose could be reduced if the symptoms were alleviated after management. Chemotherapy could be postponed if a patient’s condition still did not meet the criteria for chemotherapy treatment after adverse-event management.

Patients were followed up every 3 months until death or until the cutoff date of this study on 30 September 2012. The response rate (RR) was calculated as CR plus PR, time to progression (TTP) was defined as the period from the time of initial chemotherapy to PD and OS was defined as the period from the time of initial chemotherapy to death or the time of the final follow-up. Toxicity was assessed as grade 1 to 4 according to the US National Cancer Institute’s Terminology Criteria for Adverse Events V3.0.

### Statistical analysis

Statistical analyses were conducted using SPSS 13.0 software. Progression-free survival (PFS) was calculated from the date of randomization to the time of disease progression, death, or September 30, 2012. PFS and OS were analyzed using the Kaplan-Meier method. The chi-squared test was used to compare the differences between groups. Results with *P* <0.05 were regarded as statistically significant.

## Results

### Patients’ characteristics

Fifty-six patients were enrolled onto our study from August 2010 to September 2012. Patient characteristics are displayed in Table 1. There were 37 males and 19 females with median age 49 years (range 26 to 78) and the PS was zero or one in 50 patients. The median number of metastatic sites was two, and all of the patients presented with more than two metastatic sites. There were no statistically significant differences in the baseline characteristics between the two groups.

**Table 1:** Demographic data of Chinese patients

**Characteristic**	**Number of patients**	**%**
Median age (range)	56 (26 to 78)	
Gender		
Male	37	66.1
Female	19	33.9
ECOG performance status		
0	3	5.4
1	47	83.9
2	6	10.7
Primary tumor		
Resected	12	21.4
Unresected	44	78.6
Histology		
Diffuse	31	55.4
Intestinal	25	44.6
Sites of metastasis		
Lymph nodes	41	73.2
Liver	23	41.1
Lung	3	5.4
Peritoneum	8	14.3
Other	19	33.9

### Treatment efficacy

The 30 patients in the Ce-SOX group received an average of 3.47 cycles of chemotherapy treatment while the 26 patients in the SOX group received an average of 3.62 cycles of chemotherapy treatment. There was no statistically significant difference in the average chemotherapy cycles received by patients between the Ce-SOX and SOX groups (*P* = 0.071). The response rate in the Ce-SOX group was 54.8%, which was higher than the 44.0% in the SOX group, but failed to demonstrate statistical significance (*P* = 0.22, χ2 = 3.18). The disease-control rate of the Ce-SOX group and SOX group was 87.1% and 76.0%, respectively (*P* = 0.16, χ2 = 2.98) (Table 2).

**Table 2:** **Response rates of** c**etuximab in combination with S-1 and oxaliplatin (Ce-SOX) and S-1 and oxaliplatin (SOX) groups**

**Response**	**Ce-SOX**	**SOX**	***P***
Complete response	1	0	-
Partial response	16	11	0.11
Stable disease	10	8	0.18
Progressive disease	4	6	0.15
Response rate (%)	54.8	44.0	0.22
Disease control rate (%)	87.1	76.0	0.16

**Figure 1:**
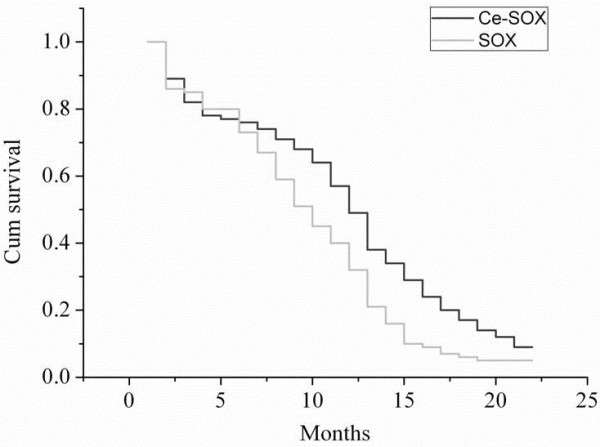
**Progression-free survival of patients in the Ce-SOX group and the SOX group estimated by Kaplan–Meier.** Cum, cumulative.

**Figure 2:**
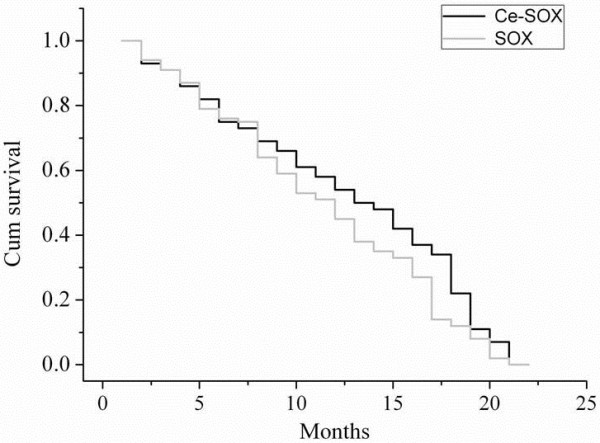
**Kaplan-Meier curve for overall survival of patients in the Ce-SOX group and the SOX group.** Cum, cumulative.

Patients were followed up for 2 years until this study ended. Median PFS in the Ce-SOX group was statistically higher than that in the SOX group (12.8 versus 10.1 months, *P* = 0.007, χ2 = 10.217) (Figure 1). The median OS of the Ce-SOX group and SOX group was 14.0 and 12.2 months, respectively (*P* = 0.043) (Figure 2). The one-year survival rate for the Ce-SOX group was 57% compared to 40% in the SOX group. The results indicated that the Ce-SOX group showed significant benefits in PFS and OS.

### Safety assessment

The observed hematological and nonhematological toxicities are shown in Table 3. The major hematological toxicities were thrombocytopenia and neutropenia. The most common grade 3/4 adverse events included bone marrow suppression, which leads to thrombocytopenia and neutropenia. There was no statistically significant difference in the incidences of adverse effects between the two groups in the hematological toxicities (Table 3). Among the nonhematological toxicities, nausea, vomiting, and diarrhea were also observed. Other recorded grade 1/2 adverse events were peripheral neuropathy, hepatic dysfunction, and renal dysfunction. There was no statistical difference in the grade 3 or 4 adverse effects between the two groups, included neutropenia (26.7 versus 34.6%), thrombocytopenia (6.6 versus 7.7%), nausea and vomiting (3.3 versus 7.7%), diarrhea (0 versus 3.8%), peripheral neuropathy (3.3 versus 0%), hepatic dysfunction (0 versus 3.8%), and hand-foot syndrome (3.3 versus 3.8%). No treatment-related deaths were recorded.

**Table 3:** Incidence of hematological and nonhematological toxicities

**Type of toxicity**	**Grade 1 to 2**			**Grade 3 to 4**		
	**Ce-SOX**	**SOX**	***P***	**Ce-SOX**	**SOX**	***P***
Hematological	22 (73.3)	17 (65.4)	0.52	10 (33.3)	11 (42.3)	0.58
Thrombocytopenia	6 (20.0)	5 (19.2)	0.94	2 (6.6)	2 (7.7)	1.0
Neutropenia	16 (53.3)	12 (46.2)	0.59	8 (26.7)	9 (34.6)	0.57
Nonhematological	16 (53.3)	21 (80.8)	0.03	3 (10.0)	5 (19.2)	0.53
Nausea and vomiting	1 (3.3)	8 (30.8)	0.008	1 (3.3)	2 (7.7)	0.59
Diarrhea	0 (0)	1 (3.8)	0.46	0 (0)	1 (3.8)	0.46
Mucositis	1 (3.3)	2 (7.7)	0.59	0 (0)	0 (0)	-
Hand-foot syndrome	2 (6.7)	1 (3.8)	0.55	1 (3.3)	1 (3.8)	1.0
Peripheral neuropathy	8 (26.7)	1 (3.8)	0.029	1 (3.3)	0 (0)	0.68
Hepatic dysfunction	3 (10.0)	2 (7.7)	0.67	0 (0)	1 (3.8)	0.72
Renal dysfunction	1 (3.3)	6 (23.1)	0.04	0 (0)	0 (0)	-

## Discussion

Gastric cancer is the second most frequent cancer in the world. Approximately 84% of gastric cancer patients will have advanced disease, and median survival of these patients without chemotherapy is only 3 to 4 months [30]. For advanced-stage gastric cancer, chemotherapy is usually the most effective treatment option. However, the 5-year survival rate for gastric cancer is just 20% [31], and the prognosis of patients with AGC is still poor. Therefore, it would be interesting to investigate new combinations and to include drugs directed against new therapeutic targets in the setting. Cetuximab, which targets human EGFR, has demonstrated promising results in treating advanced gastric cancer [1,18-22,32]. In this study, we investigated the efficacy and safety of cetuximab with the SOX regimen in advanced gastric cancer.

In first-line setting cetuximab has been evaluated in combination with different regimens of chemotherapy. Lordick *et al*. [19] found that cetuximab plus FUFOX showed an interestingly high response rate in metastatic gastric cancer. The results showed that TTP was 7.6 months (CI 95%: 5.0 to 10.1 months) and OS was 9.5 months (CI 95%: 7.9 to 11.1 months). Pinto *et al*. [21] demonstrated that the addition of cetuximab to the cisplatin/docetaxel regimen improved the ORR of the cisplatin/docetaxel doublet in the first-line treatment of advanced gastric cancer. In addition, cetuximab combined with FOLFOX4 was very active with an acceptable toxicity profile [23]. The results showed that ORR and DCR were 36.0% (95% CI = 17 to 55%) and 84.0% (95% CI = 70 to 98%), respectively. The median PFS was 6.5 months (95% CI = 5.0 to 8.0 months) and median OS was 10.6 months (95% CI = 4.4 to 16.7 months). Based on these promising results, a phase III clinical trial was conducted [33]. In the randomized, open-label, and multicenter EXPAND trial, the investigators studied the combination of capecitabine and cisplatin as a first line treatment in advanced GC and gastro-esophageal junction (GEJ) cancer with or without cetuximab. This study enrolled 904 patients with unresectable advanced GC or GEJ cancer who had not received any prior treatment with chemotherapy or radiotherapy. However, the study unfortunately did not meet the primary endpoint of an improvement in PFS (4.4 versus 5.6 months), did not increase the OS (9.4 versus 10.7 months), and showed no significant differences in RR (29 versus 30%) with and without cetuximab, respectively. Data from the EXPAND study suggested that addition of EGFR antibodies did not provide additional benefit compared with chemotherapy alone. This absence of efficacy might be due to choosing capecitabine-cisplatin as the chemotherapy backbone [33].

The SOX regimen has been evaluated in several clinical studies. Koizumi *et al*. [10] reported that SOX regimen with oxaliplatin at a dose of 100 mg/m^2^ was effective and well tolerated in AGC patients. In their study, the median PFS time was 6.5 months, the one-year survival rate was 71%, and the median survival time was 16.5 months. Moreover, a lower incidence of grade 3/4 toxicity with SOX regimen was also observed. These results indicated that SOX regimen is more tolerable and tends to be superior to S-1 plus cisplatin [8] in terms of safety. Li and Chen [34] also evaluated the combination of S-1 and oxaliplatin in a Phase II trial. In this trial, 66 patients with AGC were enrolled. Grade 3/4 anemia and neutropenia was observed in 6.4% of patients, and GI toxicities such as nausea, anorexia, and liver dysfunction were observed in 12.5%, 5.4%, and 9.7%, respectively. It was concluded from this study that the SOX regimen as a neoadjuvant treatment was associated with high efficacy and acceptable side effects. Similarly, Oh *et al*. [12] and Liu *et al*. [13] also demonstrated that the SOX regimen evidenced a relatively high response rate and was well tolerated with less toxicity and more convenient treatment as a first-line therapy for AGC patients.

In 2013, Liu *et al.*[35] conducted a trial using the SOX regimen (S-1 was administered orally, 40 mg/ (m^2^ · d), b.i.d., with a schedule of 14 days on and 7 days off, while oxaliplatin was administered 130 mg/m^2^ on day 1.) that showed there were 2 (5.26%) CR, 12 (31.58%) PR, 15 (39.47%) SD, and 9 (23.68%) PD. The overall response rate and the disease control rate (DCR) was 36.84 and 76.31%, respectively. The TTP was 7.9 months. The median survival time (MST) was 10.2 months. These results demonstrated that the SOX regimen was effective and tolerable in the treatment of patients with AGC. The similar efficacies with less toxicity of SOX regimen in the other two studies indicate that the SOX regimen has the potential to replace current regimens [36,37]. In the present study, we performed the Ce-SOX regimen and the SOX regimen (as control) for advanced gastric cancer. In the SOX regimen, the results of efficacy and safety were similar to the previous studies. In the Ce-SOX regimen, median PFS and median OS in the Ce-SOX regimen were statistically higher compared with the SOX regimen (12.8 versus 10.1 months (*P* = 0.007), 14.0 versus 12.2 months (*P* = 0.043), respectively). The one-year survival rate for the Ce-SOX group was 57% compared to 40% in the SOX group. These results suggest that cetuximab in combination with the SOX regimen shows significant therapeutic benefits to the Chinese patients with AGC. However, this study is limited by its small sample size. Future randomized, double-blind, large-scale clinical trials are warranted to clarify the role of this regimen in advanced gastric cancer.

## Conclusions

In conclusion, our study showed that cetuximab combined with the SOX regimen gave a favorable efficacy and safety profile in Chinese patients with advanced gastric cancer. Compared with the SOX regimen, this new regimen exerts significant therapeutic benefits to Chinese patients with advanced gastric cancer.

## Abbreviations

AGC: advanced gastric cancer; Ce-SOX: cetuximab in combination with S-1 and oxaliplatin; CR: complete response; DCR: disease control rate; EGFR: epidermal growth factor receptor; GC: gastric cancers; GEJ: gastro-esophageal junction; mAb: monoclonal antibody; MST: median survival time; ORRs: objective response rates; OS: overall survival; PD: progressive disease; PFS: progression-free survival; PR: partial response; PS: performance status; RECIST: Response Evaluation Criteria in Solid Tumors; RR: response rate; SD: stable disease; SOX: S-1 and oxaliplatin; TTP: time to progression.

## Competing interests

The authors declare that they have no competing interests.

## Authors’ contributions

All authors read and approved the final manuscript.

## References

[B1] Liu L Wu N Li J Novel targeted agents for gastric cancer J Hematol Oncol 2012 5 31 10.1186/1756-8722-5-31 22709792 PMC3411478

[B2] Takahashi T Saikawa Y Kitagawa Y Gastric cancer: current status of diagnosis and treatment Cancers 2013 5 48 63 24216698 10.3390/cancers5010048 PMC3730304

[B3] Wang X Wang ML Zhou LY Lu XY Yang JF Yu HG Randomized phase II study comparing paclitaxel with S-1 vs. S-1 as first-line treatment in patients with advanced gastric cancer Clin Transl Oncol 2013 15 836 842 23381898 10.1007/s12094-013-1012-6

[B4] Jemal A Bray F Center MM Ferlay J Ward E Forman D Global cancer statistics CA Cancer J Clin 2011 61 69 90 21296855 10.3322/caac.20107

[B5] Lee HH Hur H Kim SH Park AR Kim W Jeon HM Outcomes of modified FOLFOX-6 as first line treatment in patients with advanced gastric cancer in a single institution; retrospective analysis Cancer Res Treat 2010 42 18 23 20369047 10.4143/crt.2010.42.1.18 PMC2848749

[B6] Gao SG Jia RN Feng XS Xie XH Shan TY Pan LX Song NS Wang YF Ding KL Wang LD Therapeutic effects of combined oxaliplatin and S-1 in older patients with advanced gastric cardiac adenocarcinoma World J Gastroenterol 2011 17 5221 5226 22215948 10.3748/wjg.v17.i47.5221 PMC3243890

[B7] Ajani JA Rodriguez W Bodoky G Moiseyenko V Lichinitser M Gorbunova V Vynnychenko I Garin A Lang I Falcon S Multicenter phase III comparison of cisplatin/S-1 with cisplatin/infusional fluorouracil in advanced gastric or gastroesophageal adenocarcinoma study: the flags trial J Clin Oncol 2010 28 1547 1553 20159816 10.1200/JCO.2009.25.4706

[B8] Koizumi W Narahara H Hara T Takagane A Akiya T Takagi M Miyashita K Nishizaki T Kobayashi O Takiyama W Toh Y Nagaie T Takagi S Yamamura Y Yanaoka K Orita H Takeuchi M S-1 plus cisplatin versus S-1 alone for first-line treatment of advanced gastric cancer (SPIRITS trial): a phase III trial Lancet Oncol 2008 9 215 221 18282805 10.1016/S1470-2045(08)70035-4

[B9] Rosati G Ferrara D Manzione L New perspectives in the treatment of advanced or metastatic gastric cancer World J Gastroenterol 2009 15 2689 2692 19522017 10.3748/wjg.15.2689 PMC2695882

[B10] Koizumi W Takiuchi H Yamada Y Boku N Fuse N Muro K Komatsu Y Tsuburaya A Phase II study of oxaliplatin plus S-1 as first-line treatment for advanced gastric cancer (G-SOX study) Ann Oncol 2010 21 1001 1005 19875759 10.1093/annonc/mdp464

[B11] Park I Lee JL Ryu MH Chang HM Kim TW Sym SJ Lee SS Jang G Yoo C Bae KS Kang YK Phase I/II and pharmacokinetic study of S-1 and oxaliplatin in previously untreated advanced gastric cancer Cancer Chemother Pharmacol 2010 65 473 480 19551382 10.1007/s00280-009-1052-3

[B12] Oh SY Kwon HC Jeong SH Joo YT Lee YJ Cho S Kang MH Go SI Lee GW Kim H Kang JH A phase II study of S-1 and oxaliplatin (SOx) combination chemotherapy as a first-line therapy for patients with advanced gastric cancer Invest New Drugs 2012 30 350 356 20706861 10.1007/s10637-010-9507-2

[B13] Liu B Ying J Luo C Xu Q Zhu L Zhong H S-1 Combined with oxaliplatin as first line chemotherapy for Chinese advanced gastric cancer patients Hepatogastroenterology 2012 59 649 653 22328264 10.5754/hge11893

[B14] Baselga J The EGFR as a target for anticancer therapy - focus on cetuximab Eur J Cancer 2001 37 16 22 10.1016/s0959-8049(01)00233-7 11597400

[B15] Jonker DJ O’Callaghan CJ Karapetis CS Zalcberg JR Tu D Au HJ Berry SR Krahn M Price T Simes RJ Tebbutt NC van Hazel G Wierzbicki R Langer C Moore MJ Cetuximab for the treatment of colorectal cancer N Engl J Med 2007 357 2040 2048 18003960 10.1056/NEJMoa071834

[B16] Vermorken JB Trigo J Hitt R Koralewski P Diaz-Rubio E Rolland F Knecht R Amellal N Schueler A Baselga J Open-label, uncontrolled, multicenter phase II study to evaluate the efficacy and toxicity of cetuximab as a single agent in patients with recurrent and/or metastatic squamous cell carcinoma of the head and neck who failed to respond to platinum-based therapy J Clin Oncol 2007 25 2171 2177 17538161 10.1200/JCO.2006.06.7447

[B17] Ciardiello F Tortora G EGFR antagonists in cancer treatment N Engl J Med 2008 358 1160 1174 18337605 10.1056/NEJMra0707704

[B18] Moehler M Mueller A Trarbach T Lordick F Seufferlein T Kubicka S Geissler M Schwarz S Galle PR Kanzler S Cetuximab with irinotecan, folinic acid and 5-fluorouracil as first-line treatment in advanced gastroesophageal cancer: a prospective multi-center biomarker-oriented phase II study Ann Oncol 2011 22 1358 1366 21119032 10.1093/annonc/mdq591

[B19] Luber B Deplazes J Keller G Walch A Rauser S Eichmann M Langer R Höfler H Hegewisch-Becker S Folprecht G Wöll E Decker T Endlicher E Lorenzen S Fend F Peschel C Lordick F Cetuximab plusoxaliplatin/leucovorin/5-fluorouracil in first-line metastatic gastric cancer: a phase II study of the Arbeitsgemeinschaft Internistische Onkologie (AIO) Br J Cancer 2010 102 500 505 20068568 10.1038/sj.bjc.6605521 PMC2822949

[B20] Lorenzen S Schuster T Porschen R Al-Batran SE Hofheinz R Thuss-Patience P Moehler M Grabowski P Arnold D Greten T Müller L Röthling N Peschel C Langer R Lordick F Cetuximab plus cisplatin-5-fluorouracil versus cisplatin-5- fluorouracil alone in first-line metastatic squamous cell carcinoma of the esophagus: a randomized phase II study of the arbeitsgemeinschaft internistische onkologie Ann Oncol 2009 20 1667 1673 19549707 10.1093/annonc/mdp069

[B21] Pinto C Di Fabio F Barone C Siena S Falcone A Cascinu S Rojas Llimpe FL Stella G Schinzari G Artale S Mutri V Giaquinta S Giannetta L Bardelli A Martoni AA Phase II study of cetuximab in combination with cisplatin and docetaxel in patients with untreated advanced gastric or gastrooesophageal junction adenocarcinoma (DOCETUX study) Br J Cancer 2009 101 1261 1268 19773760 10.1038/sj.bjc.6605319 PMC2768436

[B22] Woll E Kuhr T Eisterer W Gattringer K Greil R Zabernigg A Hilbe W Thaler J Biweekly oxaliplatin and irinotecan chemotherapy in advanced gastric cancer. a first-line multicenter phase II trial of the Arbeitsgemeinschaft Medikamentoese Tumortherapie (AGMT) Anticancer Res 2008 28 2901 2905 19031932

[B23] Shi M Ji J Wu J Ma T Liu Y Zhou CF Su Y Ye ZB Zhang J Zhu ZG Cetuximab combined with FOLFOX4 as the first-line treatment for advanced gastric cancer: report of 25 cases from a single institution Hepatogastroenterology 2012 59 1054 1058 22281975 10.5754/hge11874

[B24] Gamboa-Dominguez A Dominguez-Fonseca C Quintanilla-Martinez L Reyes-Gutierrez E Green D Angeles-Angeles A Busch R Hermannstädter C Nährig J Becker KF Becker I Höfler H Fend F Luber B Epidermal growth factor receptor expression correlates with poor survival in gastric adenocarcinoma from Mexican patients: a multivariate analysis using a standardized immunohistochemical detection system Mod Pathol 2004 17 579 587 15073595 10.1038/modpathol.3800085

[B25] Dragovich T McCoy S Fenoglio-Preiser CM Wang J Benedetti JK Baker AF Hackett CB Urba SG Zaner KS Blanke CD Abbruzzese JL Phase II trial of erlotinib in gastroesophageal junction and gastric adenocarcinomas: SWOG 0127 J Clin Oncol 2006 24 4922 4927 17050876 10.1200/JCO.2006.07.1316

[B26] Amado RG Wolf M Peeters M Van Cutsem E Siena S Freeman DJ Juan T Sikorski R Suggs S Radinsky R Patterson SD Chang DD Wild-type KRAS is required for panitumumab efficacy in patients with metastatic colorectal cancer J Clin Oncol 2008 26 1626 1634 18316791 10.1200/JCO.2007.14.7116

[B27] Kim IJ Park JH Kang HC Shin Y Park HW Park HR Ku JL Lim SB Park JG Mutational analysis of BRAF and K-ras in gastric cancers: absence of BRAF mutations in gastric cancers Hum Genet 2003 114 118 120 14513361 10.1007/s00439-003-1027-0

[B28] Lee SH Lee JW Soung YH Kim HS Park WS Kim SY Lee JH Park JY Cho YG Kim CJ Nam SW Kim SH Lee JY Yoo NJ BRAF and KRAS mutations in stomach cancer Oncogene 2003 22 6942 6945 14534542 10.1038/sj.onc.1206749

[B29] Zhao W Chan TL Chu KM Chan AS Stratton MR Yuen ST Leung SY Mutations of BRAF and KRAS in gastric cancer and their association with microsatellite instability Int J Cancer 2004 108 167 169 14618633 10.1002/ijc.11553

[B30] Rivera F Vega-Villegas ME Lopez-Brea MF Chemotherapy of advanced gastric cancer Cancer Treatt Rev 2007 33 315 324 10.1016/j.ctrv.2007.01.004 17376598

[B31] Xu R Ma N Wang F Ma L Chen R Chen R Kebinu M Ma L Han Z Ayixiamu Mayier M Su P Naman Y Jieensi H Yang H Adili A Aili S Liu J Mayier M Su P Naman Y Jieensi H Yang H Adili A Aili S Liu J Results of a randomized and controlled clinical trial evaluating the efficacy and safety of combination therapy with endostar and S-1 combined with oxaliplatin in advanced gastric cancer Onco Targets Ther 2013 6 925 929 23926435 10.2147/OTT.S46487 PMC3728266

[B32] Amedei A Benagiano M della Bella C Niccolai E Benagiano M Bella C Niccolai E D’Elios MM Novel immunotherapeutic strategies of gastric cancer treatment J Biomed Biotechnol 2011 2011 437348 22253528 10.1155/2011/437348 PMC3255571

[B33] Lordick F Kang YK Chung HC Salman P Oh SC Bodoky G Kurteva G Volovat C Moiseyenko VM Gorbunova V Park JO Sawaki A Celik I Götte H Melezínková H Moehler M Capecitabine and cisplatin with or without cetuximab for patients with previously untreated advanced gastric cancer (EXPAND): a randomised, open-label phase 3 trial Lancet Oncol 2013 14 490 499 23594786 10.1016/S1470-2045(13)70102-5

[B34] Li T Chen L Efficacy and safety of SOX regimen as neoadjuvantn chemotherapy for advanced gastric cancer Zhonghua Wei Chang Wai Ke Z a Zhi 2011 14 104 106 21365500

[B35] Tao L Zhe L Jian Z Clinical evaluation of the combination of S-1 and oxaliplatin in the treatment of patients with advanced gastric cancer Med Innov of China 2013 10 25 26

[B36] Kang ZX Ceng HX Guo SJ Clinical curative effect observation of oxaliplatin combined S1 in treatment of 52, cases with advanced gastric cancer China Foreign Med Treat 2013 32 22 24

[B37] Fan WF Wang J Meng LJ Liu FY Pu XL Yang M Clinical study of S-1 plus oxanplatin versus S-l plus cisplatin as the first-line treatment for elderly patients with advanced gastric cancer Chinese Clin Oncol 2013 18 50 53

